# Oxidative stress in schizophrenia: a case–control study on the effects on social cognition and neurocognition

**DOI:** 10.1186/s12888-014-0268-x

**Published:** 2014-09-24

**Authors:** Cristina Gonzalez-Liencres, Cumhur Tas, Elliot C Brown, Soner Erdin, Ece Onur, Zeynep Cubukcoglu, Omer Aydemir, Aysen Esen-Danaci, Martin Brüne

**Affiliations:** Division of Cognitive Neuropsychiatry and Psychiatric Preventative Medicine, LWL University Hospital, Ruhr University Bochum, Alexandrinensr. 1-3, 44791 Bochum, Germany; Department of Psychology, Uskudar University, İstanbul, Turkey; Maryland Psychiatric Research Centre, University of Maryland School of Medicine, Baltimore, MD USA; Department of Biochemistry, Celal Bayar University, Manisa, Turkey; Department of Psychiatry, Celal Bayar University, Manisa, Turkey

**Keywords:** Neurotrophin 4, Neurotrophin 5, Glutathione, Malondialdehyde, Nitric oxide, Superoxide dismutase, Reactive oxygen species, Theory of mind, Emotion recognition, Executive functioning, Working memory

## Abstract

**Background:**

Schizophrenia is a debilitating mental disorder that presents impairments in neurocognition and social cognition. Several studies have suggested that the etiology of schizophrenia can be partly explained by oxidative stress. However, our knowledge about the implications of oxidative stress on illness-related cognitive deficits is still far from being clear. The aim of this work was to study the role of oxidative stress molecules on social cognition and neurocognition in patients with schizophrenia.

**Methods:**

We assessed the peripheral levels of several molecules associated with oxidative stress, namely nitric oxide (NO), malondialdehyde (MDA), glutathione (GSH), homocysteine, superoxide dismutase (SOD) and neurotrophin 4/5 (NT4/5), in forty–one patients with schizophrenia and forty-three healthy participants. A battery of tests to measure neurocognition and social cognition was also administered to the schizophrenia group.

**Results:**

We found that the schizophrenia group presented substantially higher levels of oxidative stress than the control group, as revealed by elevated quantities of the pro-oxidants NO and MDA, and decreased levels of the antioxidants GSH, SOD and NT4/5. Interestingly, the levels of NT4/5, which have been shown to have antioxidant effects, correlated with executive functioning, as measured by two distinct tests (WCST and TMT). However, social cognition and symptom severity were not found to be associated with oxidative stress.

**Conclusions:**

We propose a protective role of NT4/5 against oxidative stress, which appears to have a potentially beneficial impact on neurocognition in schizophrenia.

## Background

Schizophrenia is a severely debilitating mental disorder that is characterized by different neurodevelopmental and behavioral abnormalities, with deficits in perception, emotion processing and social functioning [1,2]. Multiple genetic and environmental risk factors for schizophrenia have been identified, including drug and alcohol abuse, prenatal infections and malnutrition [3-5]. Despite the heterogeneity of risk factors, the underlying biological mechanism linking these processes could be associated with oxidative stress [6-8]. Oxidative stress is induced by excessive generation of reactive oxygen species (ROS) or a lack of appropriate removal, which can consequently cause neural cell damage [9]. Oxidative stress has been found to be prevalent in many psychiatric disorders [10] and though it may not be the only cause of schizophrenia, it has been suggested to play a crucial role in the course of the illness [11,12].

Most studies that have assessed oxidative stress in schizophrenia populations point to an increase in toxic damage due to both increased pro-oxidants and decreased antioxidants [13]. In schizophrenia, numerous reports have found oxidative stress in cerebrospinal fluid (CSF) and prefrontal cortex in vivo [14], in the anterior cingulate cortex in post-mortem studies [15], and in peripheral tissues and plasma of schizophrenia patients [16-18]. More specifically, several authors have reported elevated levels of the oxidative stress indicators malondialdehyde (MDA) and nitric oxide (NO), along with lower levels of the antioxidant molecule glutathione (GSH) in schizophrenia patients as compared to healthy controls [19-21]. However, some controversy still exists regarding the levels of antioxidant enzymatic activity evaluated by superoxide dismutase (SOD), catalase (CAT) and glutathione peroxidase (GSH-Px) [11,17,22-26].

In addition to common oxidative stress biomarkers, some endogenous neurotrophic factors such as neurotrophin 4/5 (NT4/5) have recently been shown to be implicated in the up-regulation of antioxidants in neural cells [27]. While the effects of brain-derived neurotrophic factor (BDNF) on oxidative stress and its relationship to disease has been extensively studied [27], few reports have investigated how NT4/5 affects neuropsychiatric disorders. Walz and colleagues reported increased NT4/5 in the serum of patients with bipolar disorder and thus argued that the increase in NT4/5 occurs as a compensatory mechanism to deal with oxidative stress in dopaminergic neurons [28]. On the other hand, decreased antioxidant defense, including reduced levels of NT4/5, has been found in the brains of patients with Alzheimer’s disease [29-31]. However, the relationship between NT4/5 and schizophrenia remains unclear.

At the behavioral level, social functioning in schizophrenia patients is modulated by several factors, including social cognition and neurocognition [32]. Social cognition denotes the ability to process, assimilate and respond to social stimuli, and can be categorized into several domains that work at different levels; from basic low-level emotion perception, to theory of mind (i.e. to attribute and understand the mental states of others, such as beliefs and intentions) up to appropriate behavioral responses [33]. Additionally, social cognitive deficits have been linked to symptom severity in schizophrenia, and are negatively associated with quality of life and social functioning [34]. Neurocognition, on the other hand, is defined by the cognitive skills necessary to carry out psychological processes, and can be categorized into independent but interrelated subdomains, including executive function, working memory and attention. Intact neurocognition is essential for social functioning [35,36].

Several studies suggest that oxidative stress has detrimental effects on socio- and neurocognitive abilities in schizophrenia. For instance, Pavlovic and colleagues have suggested that oxidative stress is increased in patients with greater positive symptoms [21]. Equally interesting, another study showed in post-mortem studies that schizophrenia patients had higher oxidative stress in the anterior cingulate cortex (ACC), a brain region involved in emotion and social processing [15]. Most notably, Martinez-Cengotitabengoa and colleagues were the first to investigate the potential relationship between the levels of oxidative stress and neurocognition in schizophrenia, and found a significant correlation between GSH and executive function [37]. Together, these studies point toward a general effect of oxidative stress on social cognition and neurocognition. Despite the extensive studies focusing on oxidative stress indicators as possible biomarkers, there are no reports that have systematically explored the relationship between oxidative stress, social cognition and neurocognition in schizophrenia.

The present study aimed to first quantify and compare markers of oxidative stress in a schizophrenia sample and a healthy control group, and then to closely examine the relationship between oxidative stress parameters and a variety of social and cognitive domains in schizophrenia. We were mostly interested in the link between the levels of several biomarkers (namely SOD, GSH, MDA, homocysteine, NO and NT4/5) and theory of mind (ToM), emotion processing, working memory and executive functioning. We hypothesized that (1) oxidative stress is higher in the schizophrenia patients and (2) is related to deficits in social cognition (i.e. emotion processing and ToM) and neurocognition (executive functioning and working memory).

## Methods

### Participants

Fifty clinically stable schizophrenia patients and fifty age-matched healthy controls without psychiatric disorders, as evaluated by structured clinical interview for DSM-IV (SCID), were recruited from Celal Bayar University, Psychiatry Department, Psychosis Unit. The schizophrenia group was diagnosed according to the DSM-4TR by clinically trained and experienced psychiatrists. The inclusion criteria were as follows: age between 18–60, no evidence of neurological deficits, no drug or alcohol abuse and no hospitalization in the six months before the recruitment phase of the study. After the recruitment, all patients and controls were screened for chronic physical illness (history of hypertension, diabetes, hyperlipidemia, peripheral vascular disease, coagulopathy and the presence of hematological, renal, or hepatic disease) from their previous medical records. Due to the potential confounding effects on peripheral markers of oxidative stress, participants who had evidence of chronic physical illness were excluded, according to consultation with an internal medicine specialist. Notably, none of the patients or controls were receiving anti-inflammatory medications or vitamin supplements on a regular basis, as evaluated by the sociodemographic questionnaires. This screening procedure was done before data collection, and consequently a total of forty-one schizophrenia patients and forty-three healthy controls were included in the final sample for the study. The socio-demographical and clinical features of the sample are summarized in Table 1. Chlorpromazine equivalents were calculated for all patients in order to control for medication effects [38,39]. As a note, all patients were treated with second and third generation antipsychotics (14.6% olanzapine, 14.6% quetiapine, 34% risperidone, 22% aripiprazole, 22.2% clozapine, 9.8% others (sertindole, paliperidone, ziprasidone)). The study was approved by the Institutional Review Board (IRB) at the Celal Bayar University and was performed in accordance with the Declaration of Helsinki. All participants were evaluated for their capacity to give informed consent and provided written informed consent after all procedures were fully explained.

**Table 1:** **Demographic, clinical and cognitive variables**

		**Schizophrenia**			**Controls**			
		**Percentage (Count)**	**Mean**	**SD**	**Percentage (Count)**	**Mean**	**SD**	**Statistics**
**Sex**	**Males**	46% (19)			42% (17)			X^2^(2,N = 84) = .39, p = .53
	**Females**	54% (22)			58% (26)			
**Age (years)**			32.32	9.08		29.74	8	t(82) = 1.38, p = .17
**Education (years)**			11.06	3		11.14	2.12	t(82) = .142, p = .57
**Nr. of Hospitalization**			1.5	1.99				
**Duration of illness (years)**			9.42	6.29				
**CPZ equivalents (mg)**			457.55	280.94				
**PANSS Positive subscale**			11.96	4.67				
**PANSS Negative subscale**			17.91	6.15				
**PANSS General subscale**			28.39	8.72				
**PANSS Total**			58.26	17.82				
**Memory Quotient**			74,05	33.48				
**WCST Correct Responses**			64.97	21.49				
**WCST Completed Groups**			2.44	2.13				
**RVLT (learning score)**			5.31	2.08				
**TMT- A (seconds)**			56.60	29.52				
**TMT- B (seconds)**			130.09	71.96				
**Hinting (total correct)**			12.11	3.37				
**FRT (total correct)**			11.19	2.65				

PANSS: Positive and Negative Syndrome Scale; WCST: Wisconsin card sorting test; RVLT: Rey verbal learning test; TMT: Trail making test; FRT: Face Recognition Test.

### Symptomatology (PANSS)

Symptom severity was measured with the Positive and Negative Syndrome Scale (PANSS [40]), a 30-item semi-structured interview designed to assess three symptom categories associated with schizophrenia: positive symptoms (e.g., hallucinations and delusions), negative symptoms (e.g., avolition and anhedonia), and general symptoms (e.g., hostility, and depression). One trained and calibrated rater assigned a score from 1 to 7 for each item, with higher scores indicating more severe psychopathology. The patient group mean scores for the PANSS were 11.96 ± 4.67 for positive symptoms, 17.91 ± 6.15 for negative symptoms, 28.39 ± 8.72 for general symptoms, and 58.26 ± 17.82 for the total score.

### Cognitive assessment

Cognitive assessment (i.e. neurocognition and social cognition) was only administered to the schizophrenia group, and not to the healthy controls, on the same day as the blood sample collection.

### Neurocognitive assessment

The Turkish versions of two neuropsychological tests were used to evaluate intelligence, memory and executive functioning. The tests were administered and scored by a trained psychiatrist (Z.C.) who was blind to the clinical data of the patients. More specifically, working memory performance was assessed with the Wechsler Memory Scale-III (WMS-III; [41]). Age-corrected indices of the Memory Quotient were accepted as a measure of working memory performance. Verbal learning was measured by the Rey Auditory Verbal Learning Test [42]. The learning score was calculated from the total learning on trials 1 to 5. Executive functioning was measured with the Wisconsin Card Sorting Test-CV64 (WCST; [43]), where number of correct answers, number of perseverative errors and number of completed groups were included in the analyses. Visuo-spatial orientation was measured with the number of correct responses in the Judgment of Line Orientation Test [44]. Visual search, scanning, speed of processing and mental flexibility was measured by the total amount of time (seconds) to complete the Trail Making Test A and B (TMT A and B) [45].

### Social cognitive assessment

The Turkish versions of three widely used social cognition measures were used to evaluate emotion perception and theory of mind skills. The performance tests were administered by a trained psychiatrist and then patients were rated by a psychiatrist and a psychologist together to improve accuracy and to rule out potential interrater differences. More specifically, emotion perception was measured using the Face Emotion Identification Task (FEIT) and the Face Emotion Discrimination Task (FEDT) [46] using six basic facial emotion expressions (happy, angry, afraid, sad, surprised, and ashamed) presented on screen. Cognitive theory of mind (ToM) abilities were measured with the Hinting task [47], which consists of ten brief written vignettes describing social scenarios that the participant is asked to interpret. The total number of correct responses ranges from 0 to 20, with higher scores indicating better performance.

### Assessment of oxidative stress molecules

Patients were asked to refrain from eating or doing physical exercise 8 hours before the beginning of the sample collection. Blood samples (10 mL) were collected from the participants at admission time (9:00 a.m. to 10:00 a.m.) and were placed in three tubes: one without anticoagulant for serum and two with 0.1 mL of 0.47 mol/L EDTA (anticoagulant), one of which was utilized for plasma and one for whole blood analysis. Plasma and serum were obtained by centrifugation and together with the whole blood sample were kept at −80°C until analysis. Neurotrophin-4/5 (NT4/5), superoxide dismutase (SOD), nitric oxide (NO), and homocysteine were analyzed from serum, malondialdehyde (MDA) from plasma and total glutathione (GSH) from whole blood samples.

SOD activity was determined using enzyme-linked immunosorbent assay (ELISA), which measures the three types of SOD (Cu/Zn, Mn, and FeSOD), according to the manufacturer’s instructions (Cayman Chemical Company, Michigan, USA). The working principle of the kit consists of the utilization of tetrazolium salt to detect the dismutation of superoxide radicals produced by xanthine oxidase and hypoxanthine. One unit of SOD corresponds to the amount of enzyme required for 50% of the dismutation of superoxide radical. Absorbance was read at 450 nm.

Serum NO levels were assayed by the Griess method. The Griess diazotization reaction detects nitrite created by the spontaneous oxidation of NO. Samples are incubated in an acidic environment with sulfanilamide and N-1-naphthyl ethylene diamine (NED), which convert the nitrites into a purple product. Absorbance was read at 550 nm.

NT4/5 levels from serum samples were measured by ELISA according to the manufacturer’s protocol (Cusabio Biotech, Wuhan, China). We deposited the samples on microplates coated with antibodies specific for NT4/5. After washing the sample and leaving NT4/5 bound to the immobile antibody, a biotin-conjugated antibody was added to the microplates, followed by another wash and the addition of avidin-conjugated horseradish peroxidase (HRP). After washing, a substrate was added and color developed, which we measured spectrophotometrically at 450 nm.

Serum homocysteine levels were determined by chemiluminescence automatic immunoassay analyzer as indicated by the manufacturer’s protocol (Immulite-2000, Diagnostic Products Corporation, LA, USA). A chemiluminescent substrate was added to a reaction tube where homocysteine was bound to a coated bead. The signal is generated in proportion to the bound enzyme.

GSH and MDA levels were determined by HPLC according to the manufacturer’s instructions (ImmuChrom GmbH Schimadzu Prominence, Kyoto, Japan). Analytical RP-HPLC separations were performed using a Shimadzu Prominence HPLC system equipped with a Shimadzu SPD-20A detector (Kyoto, Japan).

### Statistical analyses

The assumption criteria for conducting parametric statistical analyses were checked for in the preliminary step. The distribution of the outcome and dependent variables was checked with the Shapiro-Wilk test. Non-parametric tests were utilized where normality was violated. The patient and control group differences in sociodemographics were tested with independent t-tests and chi-square. Group differences in oxidative stress molecules were tested using Mann Whitney U tests. A Bonferroni-corrected critical p value was calculated based on the total number of group comparisons to adjust the level of significance for multiple comparisons (critical P = .009). Lastly, Pearson correlation analyses were performed between the social and neurocognitive measures and the oxidative stress molecules in the schizophrenia patients. Statistical significance was set to p <0.05. All statistical analyses were performed with SPSS version 20.

## Results

### Group comparison of the levels of oxidative stress related molecules

We found significant group differences in all oxidative stress-related molecules except for plasma homocysteine levels (F (1,83) = .002, p = 0.965). Accordingly, higher levels of MDA and NO were observed in the schizophrenia group. In addition, significantly lower levels of GSH, SOD and NT4/5 were found in the schizophrenia patients. The group differences and the statistical values are summarized in Table 2.

**Table 2:** **Group means for markers of oxidative stress and results of group comparisons**

	**Schizophrenia**		**Controls**		**Statistics**		
					**(Mann Whitney U)**		
	**Mean**	**SD**	**Mean**	**SD**	**U (df)**	**Z**	**p**
Homocystein (μmol/L)	16.52	9.73	16.62	12.22	845.5(82)	.27	0.775
GSH (μmol/L)	539.46	206.58	986.23	179.68	105.0(82)	6.95	<.001
MDA (μmol/L)	1.19	0.17	0.98	0.22	359.0(82)	4.68	<.001
NO (μmol/L)	92.15	37.55	65.23	39.53	509.0(82)	3.33	0.001
SOD (U/ml)	0.26	0.15	0.39	0.16	177.5(82)	6.30	<.001
NT4/5 (ng/ml)	5.2	2.88	6.56	2.79	620.5(82)	2.34	0.02

GSH: total glutathione; MDA: malondialdehyde; NO: nitric oxide; SOD: superoxide dismutase; NT4/5: neurotrophin 4/5.

### Correlation analyses

**Figure 1:**
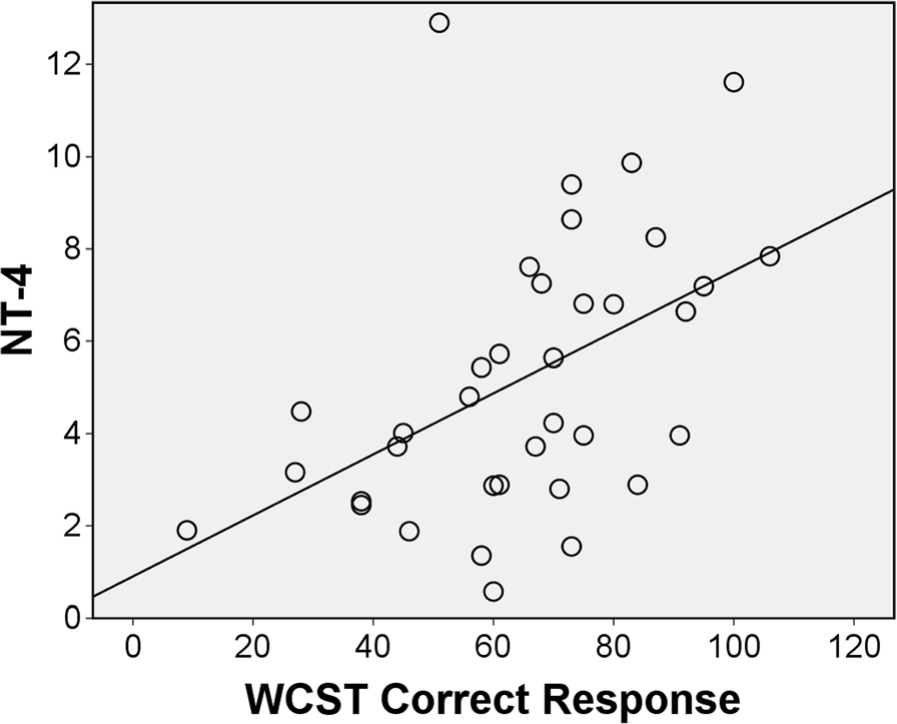
**Scatterplot between NT4/5 and executive function (WCST correct responses).**

SOD levels were significantly associated with GSH levels (r = 0.349; p = < .05). Although not significant, consistent findings were observed between pro-oxidant and antioxidant molecules such that antioxidants showed negative associations with pro-oxidants (Table 3). In regards to the association of social cognition and neurocognition with these oxidative stress markers, we found a significant correlation between NT4 levels and executive functioning, as shown by the number of correct responses (r = .474, p < .001) and completed groups (r = −.399, p < .001) in the WCST (Figure 1). In addition, a negative correlation (r = −0.333, p = .047) was observed between the total time to complete the TMT-A and plasma NT4 levels in the schizophrenia patients. However, social cognition measures were not correlated with any of the oxidative stress indicators. We also did not find any correlations between the oxidative stress markers and medication, duration of illness nor symptom severity measured with the PANSS. The correlation analyses are summarized in Table 3.

**Table 3:** **Correlations between oxidative stress markers and measures of social and neurocognition in schizophrenia patients**

	**Homocystein**	**GSH**	**MDA**	**NO**	**SOD**	**NT4/5**
Homocystein	-					
GSH	.250	-				
MDA	.111	.175	-			
NO	-.121	-.047	-.189	-		
SOD	-.098	.346*	-.179	.038	-	
NT4/5	.233	.213	.098	-.240	-.011	-
Hinting Task	-.205	-.063	.198	-.030	-.064	.043
Face Recognition Test	.310	-.152	.102	.132	-.228	.083
RVLT	.032	-.171	.071	-.201	-.001	.092
WCST Correct response	.260	.001	-.071	.120	-.051	.474**
WCST Completed groups	.195	.171	.171	-.131	-.218	.399*
MQ	-.097	-.054	.216	-.062	.041	.269
TMT-A	-.158	-.135	-.147	.150	.044	-.333*
TMT-B	-.251	-.119	-.117	.130	.048	-.272

GSH: total glutathione; MDA: malondialdehyde; NO: nitric oxide; SOD: superoxide dismutase; NT4/5: neurotrophin 4/5; WCST: Wisconsin Card Sorting Test; MQ: Memory Quotient; RVLT Rey verbal learning test; MQ: memory quotient; TMT: Trail Making Test. *p = 0.05, **p = 0.01. Values in table represent correlation coefficients.

## Discussion

The present study sought to compare the levels of oxidative stress between schizophrenia patients and healthy controls, and to examine the relationship between oxidative stress parameters (including NT4/5), social cognition and neurocognition in the patient group. We found higher levels of oxidative stress in schizophrenia patients as compared to controls, as well as a correlation between NT4/5 and neurocognition but not social cognition in the clinical group. This highlights the relationship between biological markers and cognition in neuropsychiatric disorders.

Numerous studies have previously assessed the levels of oxidative stress biomarkers in schizophrenia patients, and most have pointed towards an imbalance in the reduction-oxidation (redox) system due to increased pro-oxidants and reduced antioxidants [12,20-22]. In agreement with previous findings, we show that the schizophrenia group exhibits significantly higher levels of MDA and NO [20,23,48], as well as lower quantities of SOD and GSH [20,22,23,49], both of which support the hypothesis that these compounds contribute to the imbalance between the generation and removal of ROS. Compatible with these findings, supplementation with essential polyunsaturated fatty acids (which are decreased due to lipid peroxidation), Vitamin C and Vitamin E (two antioxidants) improves symptomatology, general psychopathology and quality of life in schizophrenia patients [50].

On the other hand, our findings on the homocysteine levels between groups contradicts previous studies showing an increase of homocysteine in the schizophrenia group [51,52]. This contrast in outcomes could be due to differences in the age and duration of illness of the participants used in other samples, as well as the potential variability caused by the different methods used to assess the levels of this compound [51,52]. Alternatively, our results may also suggest a non-specific influence of homocysteine on the neurodevelopment of schizophrenia.

In addition, we observed a significant correlation between the levels of GSH and SOD, which may indicate a potential connection between these two antioxidants. Moreover, we found a trend that suggests a positive relationship between GSH and NT4/5, whereas NO appears to be inversely related to NT4/5, although these correlations did not reach statistical significance. This is compatible with the association between increased NO and oxidative stress, while GSH and NT4/5 are protective against oxidative damage [27,53-58]. In support of this relationship, a study showed that adding NT4/5 to cultured midbrain neural cells prevented them from dying due to NO-dependent oxidative damage [59]. It is plausible that a similar mechanism occurs in humans; nonetheless, further studies are needed to assess this possibility.

One main finding of this work is the presence of decreased NT4/5 levels in the schizophrenia group. Some authors have found that, although NT4/5 has protective effects in oxidative stress-induced apoptosis *in vitro*, NT4/5 can potentiate unprogrammed cell death [56,57,60]. Accordingly, it has been previously suggested that apoptotic vulnerability is a characteristic of schizophrenia, even though neuronal apoptosis does not seem to occur more often than in healthy controls [61]. Other studies have found that apoptotic signaling molecules were higher in a schizophrenia group, but there was no difference in the apoptotic trigger molecules (i.e. caspase 3) when compared to a healthy group [61,62]. They therefore proposed that apoptotic vulnerability is increased in schizophrenia and presumably leads to local synaptic loss but not cell death. Taken together, our results may then indirectly suggest that schizophrenia patients are more vulnerable to apoptotic cell death, and that NT4/5 is a protective regulator of such cellular damage caused by oxidative stress. The mechanism underlying this process could involve the up-regulation of antioxidants by NT4/5 [27]. While no studies have so far investigated the relationship between NT4/5 and oxidative stress in schizophrenia, Walz and colleagues found increased levels of NT4/5 in bipolar patients, which the authors justified as being a compensatory mechanism against oxidative damage in dopaminergic neurons [28]. It is likely that, due to the lower NT4/5 levels, this compensation mechanism is not possible in schizophrenia. Future research may profit from focusing on how NT4/5 impacts on cognition.

Interestingly, we found a positive correlation between NT4/5 and executive functioning as assessed by the WCST, as well as a positive correlation between NT4/5 and visual attention and task switching as tested by the TMT-A, which implies that NT4/5 has some residual beneficial effects on neurocognition in this disease. It is possible that NT4/5 acts as a shielding factor by decreasing oxidative status and thus protects neuronal synapses, which in turn facilitates better functioning. Although not widely investigated, this is in line with studies suggesting a relationship between oxidative stress and neurocognition in a variety of other clinical populations [63-66]. Nevertheless, there is only one study in the literature that examined the relationship between oxidative damage and neurocognition in schizophrenia. In this study, Martinez-Cengotitabengoa and colleagues found a positive correlation between GSH and executive functioning, and a negative correlation between nitrites (the final product of NO) and executive functioning in first episode psychosis patients (FEP) [37]. However, the results from our study did not confirm these previous findings. It is possible that methodological differences and the duration of illness, as the study from Martinez-Cengotitabengoa et al. used FEPs, led to these divergent results. In fact, a recent meta-analysis has suggested that a number of oxidative stress parameters, including nitrites, are dependent on the illness state, i.e. the peripheral amounts depend on whether the patients are in their first episode of psychosis, acutely relapsed inpatients or stable outpatients [67]. Further work is required to assess the progression of, and the relationship between, these and other biomarkers and neurocognition in schizophrenia.

Contrary to our hypothesis, we did not find a relationship between oxidative stress biomarkers and social cognition. Although neurocognitive deficits in schizophrenia explain some variance of social cognitive deficits, a substantial number of studies have demonstrated the independent role of social cognition in symptom severity, social functioning and quality of life in schizophrenia patients (reviewed in [33]). Furthermore, many studies have successfully identified a separate function for different subdomains of social cognition [68]. However, the distinction between the domains of social cognition and the associated brain areas is less clear when compared to similar work on neurocognition. For instance, neuroimaging data on neurocognition suggests a specific task-dependent activation of brain regions, such as in the dorsolateral pre-frontal cortex (DLPFC), while performing a working memory task [69]. Taken together with postmortem studies in schizophrenia [70,71], it is plausible to argue that the damage caused by oxidative stress to specific brain regions may negatively influence neurocognitive faculties.

In contrast, activity in a substantially broader network involving the cingulate cortex, temporoparietal junction (TPJ), inferior parietal cortex (IPC), DLPFC and ventromedial pre-frontal cortex (VMPFC) has emerged for emotion recognition and theory of mind-related social cognitive tasks [72]. Thus, a similar proposal might not be as legitimate for social cognition, especially as previous studies have shown that neural compensation for damage to the brain network related to social cognitive tasks is possible. For instance, Newsome et al. demonstrated that a group of adolescents who had traumatic brain injuries (TBI) recruit alternative neural pathways to compensate for theory of mind impairments related to the affected areas by TBI, and hence had similar processing speeds as healthy controls [73]. This evidence may provide a clue to the interpretation of the result that we did not find any correlation between social cognition and oxidative stress, even though the NT4/5 levels that were negatively associated with NO predicted some variance in executive functioning. Lastly, it should also be noted that our work involved the most commonly studied subdomains of social cognition, namely emotion recognition and theory of mind. However, other social cognitive domains such as attributional style and metacognition should also be taken into consideration in future studies examining the effects of oxidative stress on cognitive domains.

This study presents some limitations. Firstly, the relationship between neurocognition and social cognition and oxidative stress markers were not evaluated in the healthy group. Therefore, our findings are more specific to a schizophrenia population and it is not clear whether or not the relationship between oxidative stress and neurocognition is specific to patients with this disorder. Secondly, although we carefully controlled for the effects of chronic physical illness on oxidative stress molecules, nearly all of the schizophrenia patients (87,8%; N = 36) were smokers, which was not the case for the controls, and therefore the effects of smoking on oxidative stress molecules [74] cannot be ruled out. We also did not consider body mass index (BMI), which may affect oxidative stress status [75]. Finally, this work was carried out utilizing peripheral markers of oxidative stress and it is thus possible that the amount of these molecules does not accurately reflect central levels.

## Conclusion

In conclusion, our study confirms that the detrimental effects of oxidative damage in schizophrenia are due to a combination of reduced protection and augmented oxidative insults. However, oxidative parameters should not be used as biomarkers for schizophrenia given that these are not disease specific, although they might have potential as markers to determine cognitive faculties. Interestingly, our results bring new insight into the relationship between NT4/5 and neurocognition in schizophrenia. This study provides further clues to the underlying role of pathological differences in oxidative stress and how this translates to deficits at the behavioral level, with NT4/5 being of particular interest here.
